# Cardio-pulmonary parasites of the European wildcat (*Felis silvestris*) in Germany

**DOI:** 10.1186/s13071-022-05578-z

**Published:** 2022-12-05

**Authors:** Katrin Bisterfeld, Marie-Kristin Raulf, Patrick Waindok, Andrea Springer, Johannes Lang, Michael Lierz, Ursula Siebert, Christina Strube

**Affiliations:** 1 Institute for Parasitology, Centre for Infection Medicine, University of Veterinary Medicine Hannover, Buenteweg 17, 30559 Hanover, Germany; 2 Institute for Terrestrial and Aquatic Wildlife Research, University of Veterinary Medicine Hannover, Werftstrasse 6, 25761 Buesum, Germany; 3 Clinic for Birds, Reptiles, Amphibians and Fish, Justus-Liebig-University Giessen, Frankfurter Strasse 114, 35392 Giessen, Germany

**Keywords:** *Aelurostrongylus abstrusus*, *Troglostrongylus brevior*, *Angiostrongylus chabaudi*, *Capillaria aerophila*, Lungworms, Feline Lungworms, Prevalence, Epidemiology

## Abstract

**Background:**

In the last years, research on feline cardio-pulmonary parasites has considerably increased in Europe. Not only domestic cats (*Felis catus*), but also European wildcats (*Felis silvestris*) can serve as definitive hosts for these nematodes. The *F. silvestris* population in Germany has been growing rapidly within the last decades; therefore, the assessment of its cardio-pulmonary parasite status is of importance to unravel whether the wildcat population serves as a substantial reservoir for these nematodes and might pose a health threat to domestic cats.

**Methods:**

As part of a nature conservation project for European wildcats in the German federal state Rhineland-Palatinate, lungs (*n* = 128) and hearts (*n* = 111) of 128 *F. silvestris* found dead were examined for cardio-pulmonary parasites. All isolated parasites were identified morphologically, and results were confirmed by molecular analysis of a total of 3–11 specimens of each worm species.

**Results:**

A total of 70.3% (90/128) wildcats were positive for at least one lungworm species. *Angiostrongylus chabaudi* was most common (53.1% [68/128]), followed by *Aelurostrongylus abstrusus* (42.2% [54/128]), *Troglostrongylus brevior* (31.3% [40/128]) and *Capillaria* spp. (3.1% [4/128]). Of note, about two-thirds of the infected wildcats harboured coinfections. Infection intensities ranged from 1 to 167 nematodes per wildcat. Generalised linear models revealed a strong correlation between *A. chabaudi* and *A. abstrusus* infection, and prevalences were higher in adult than in younger wildcats, except for *T. brevior*. Moreover, the *T. brevior* prevalence varied significantly with nutritional status.

**Conclusions:**

This study shows that feline cardio-pulmonary nematodes are common parasites in European wildcats in Germany but do not appear to have a serious impact on the overall health of the population. Due to presumed spillover events via prey, cardio-pulmonary nematodes may circulate between the wildcat population and domestic cats and might therefore pose a health risk to individual domestic cats.

**Graphical abstract:**

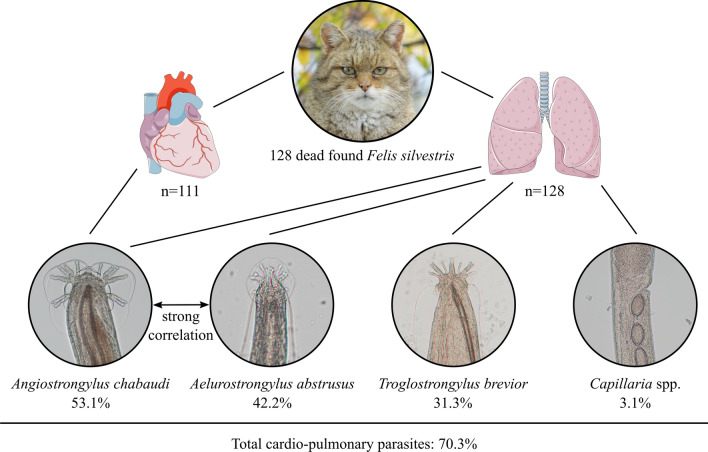

**Supplementary Information:**

The online version contains supplementary material available at 10.1186/s13071-022-05578-z.

## Background

The population of the European wildcat (*Felis silvestris*) has suffered from habitat destruction and fragmentation as well as hunting in the past, leading to their near eradication in the early twentieth century. Therefore, numerous protective measures have been established since 1922, enabling the recovery of the population until today [1]. Even though listed as Least Concern in the IUCN Red List of Threatened Species in 2021 [2], the European wildcat still belongs to the group of strictly protected species in Germany (Section 7 (2) sentence 14 of the Federal Nature Conservation Act, *Bundesnaturschutzgesetz* [3]; Council directive 92/43/EEC, Annex IV [4]). In Germany’s Red List [5], *F. silvestris* is classified as Threatened (equals Vulnerable of the IUCN Red List), although various nature conservation projects, e.g. of the German Federation for the Environment and Nature Conservation, Friends of the Earth Germany (*Bund fuer Umwelt und Naturschutz Deutschland, BUND*), have been promoting its re-occurrence in Germany in recent decades. As part of these projects, conservation measures contribute to the preservation and population increase of the European wildcat.

Wildlife predators, such as *F. silvestris*, often serve as a reservoir for manifold parasites, which may have detrimental effects on the populations’ health or can be transmitted to domestic animals and humans. Thus, parasite surveillance is important to evaluate the need for further conservation measures and to estimate the risk of spillover events to domestic and synanthropic cycles in terms of the One Health concept. Regarding parasites, cardio-pulmonary nematodes might constitute a health threat to both European wildcats and domestic cats (*Felis catus*).

The feline-specific metastrongyloids *Aelurostrongylus abstrusus*, *Troglostrongylus brevior* and *Angiostrongylus chabaudi* settle in the cardio-pulmonary system. The life cycles of *A. abstrusus* and *T. brevior* exhibit similar features with first-stage larvae (L1) excreted by the feline definitive hosts via the faeces. Various gastropods, such as slugs, serve as intermediate hosts in which third-stage larvae (L3) develop [6]. Ingestion of infective L3 by the definitive felid host occurs through ingestion of intermediate hosts or, more likely, paratenic hosts such as small rodents, birds or other vertebrates [7–9]. Additionally, potential lactogenic transmission of *T. brevior* to the offspring has been suggested in domestic cats [10, 11]. By contrast, *A. chabaudi* is less studied; therefore, its life cycle remains largely unknown. However, it is hypothesised to be similar to other metastrongyloids with excretion of L1 via faeces and further development in gastropods as intermediate hosts [12]. Adult metastrongyloids differ in size indicating their localisation in the lung and/or heart of their definitive host. Adult *A. abstrusus* are the smallest common feline metastrongyloids with 5–10 mm length and 60–80 µm width [13], inhabiting the alveolar ducts and bronchioles [14]. Adult *T.* *brevior* are larger (5–13 mm long, 0.3–0.4 mm wide) and settle in the bronchi and bronchioles [13], while adult *A. chabaudi* (14–22 mm in length, 0.2–0.3 mm in width [12]) are located in the right heart and pulmonary arteries of their definitive host [15].

The trichuroid nematode *Capillaria aerophila* (syn. *Eucoleus aerophilus*) is another important lungworm of felines. Unlike metastrongyloids, eggs are excreted via faeces into the environment, where the infective L1 develops within the egg. Wildcats can acquire infections by ingesting infective eggs or earthworms, which are suspected to be facultative intermediate or paratenic hosts [16]. Adult worms, which are 20–30 mm long and 60–180 µm wide [17], are located beneath the mucosa of the larger air ducts, i.e. trachea, bronchi and bronchioles [18].

While the occurrence of lungworms in *F. silvestris* is well studied in other European countries, mainly Italy, Greece and Romania [19–23], knowledge regarding their prevalence in Germany is limited. In two previous German studies, the mentioned lungworms were already detected in *F. silvestris* [24, 25], demonstrating the presence of various cardio-pulmonary parasites in European wildcats in Germany. However, the sample size of these previous studies was rather low (5 and 15 wildcats, respectively), so representative data on the prevalence of these parasites are not yet available. Thus, the aim of this study was to survey cardio-pulmonary parasites in *F. silvestris* in Germany to obtain a comprehensive picture of infection extensities and intensities, which helps to assess the potential role of wildcats as a lungworm reservoir for domestic cats.

## Methods

### European wildcat samples

**Fig. 1:**
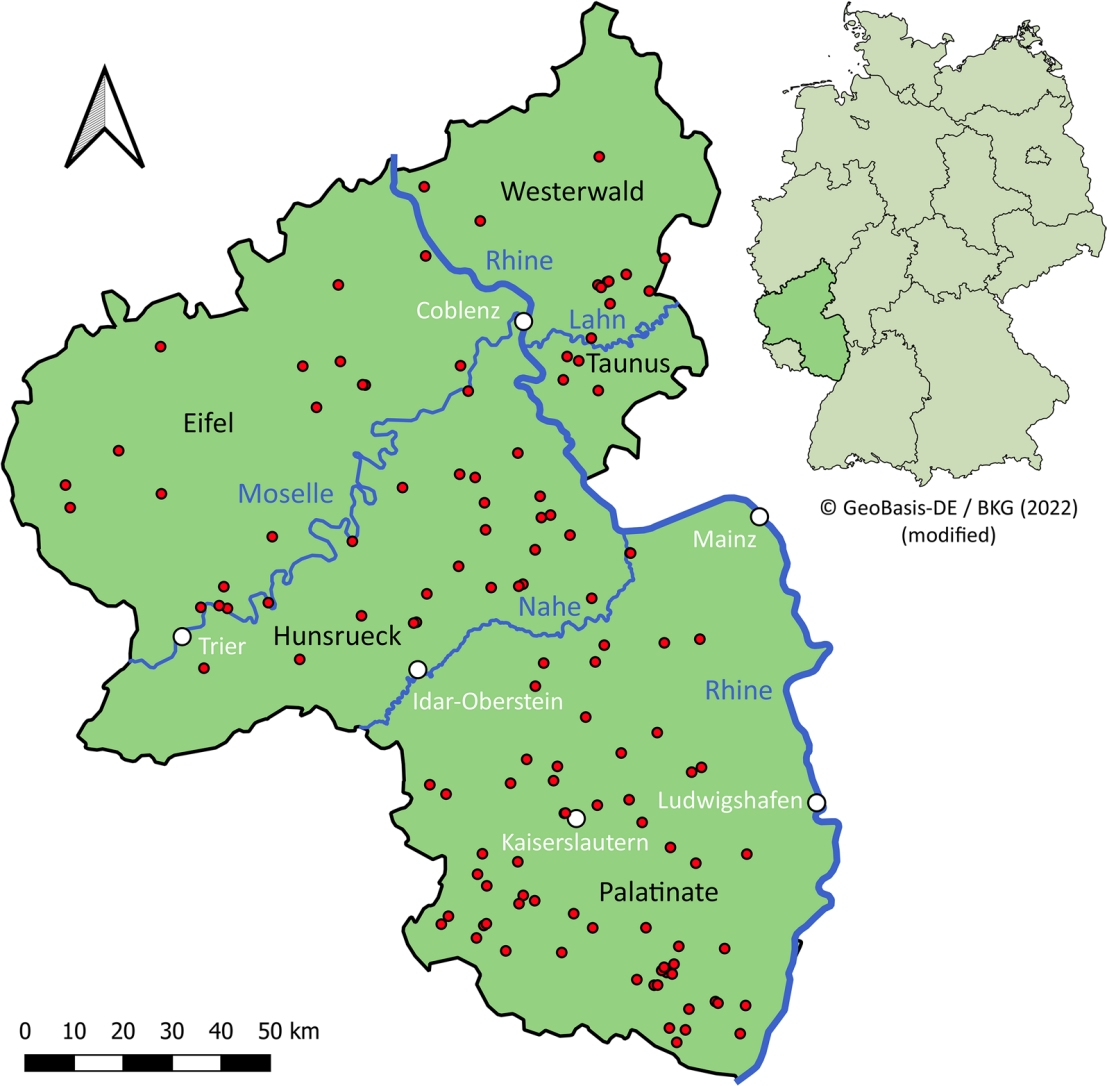
Map of the German federal state Rhineland-Palatinate showing the sampling locations (red dots) of dead found European wildcats. The scale bar represents 50 km and the arrow indicates a northern orientation. The map of Rhineland-Palatinate was created using QGIS version 3.22.2 [58]. The map of Germany was obtained from GeoBasis-DE/BKG [59]

The carcasses of 128 *F. silvestris* were available from the project “Monitoring of dead wildcats in Rhineland-Palatinate (*Totfundmonitoring Wildkatze in Rheinland-Pfalz*)” executed by the German Federation for the Environment and Nature Conservation, Friends of the Earth Germany, state association of Rhineland-Palatinate [*Bund fuer Umwelt und Naturschutz Deutschland* (*BUND*), *Landesverband Rheinland-Pfalz*]. The specimens were found dead from 2018 to 2020 by citizens in the German federal state Rhineland-Palatinate, reported to the BUND and collected by volunteer helpers (Fig. 1). Dissections were carried out by the BUND and the cooperating institutions Will and Liselott Masgeik Foundation, OEKO-LOG field research and the Clinic for Birds, Reptiles, Amphibians and Fish, Justus-Liebig-University Giessen [26]. The species *F. silvestris* was verified by morphometric and genetic (reduced single nucleotide polymorphism panels [27]) examination as previously reported [26]. During dissection, different parameters such as sex, age, nutritional condition and state of decomposition were assessed as part of the BUND project [26]. In the presented study, the nutritional condition was classified based on different fat deposits as described by Leonhardt et al. [26] with slight modifications: wildcats were classified into a ‘very bad’ nutritional condition if both subcutaneous and visceral fat as well as kidney or coronary fat was not present. Furthermore, the factors ‘very bad’ and ‘cachectic’ were summarised because of missing data on coronary fat for some of the wildcats. The classification scheme is given in Additional file 1. Of the 128 carcasses, 128 lungs and 111 hearts were available and stored at −20 °C until parasitological examination.

### Screening for cardio-pulmonary parasites and morphological identification

**Fig. 2:**
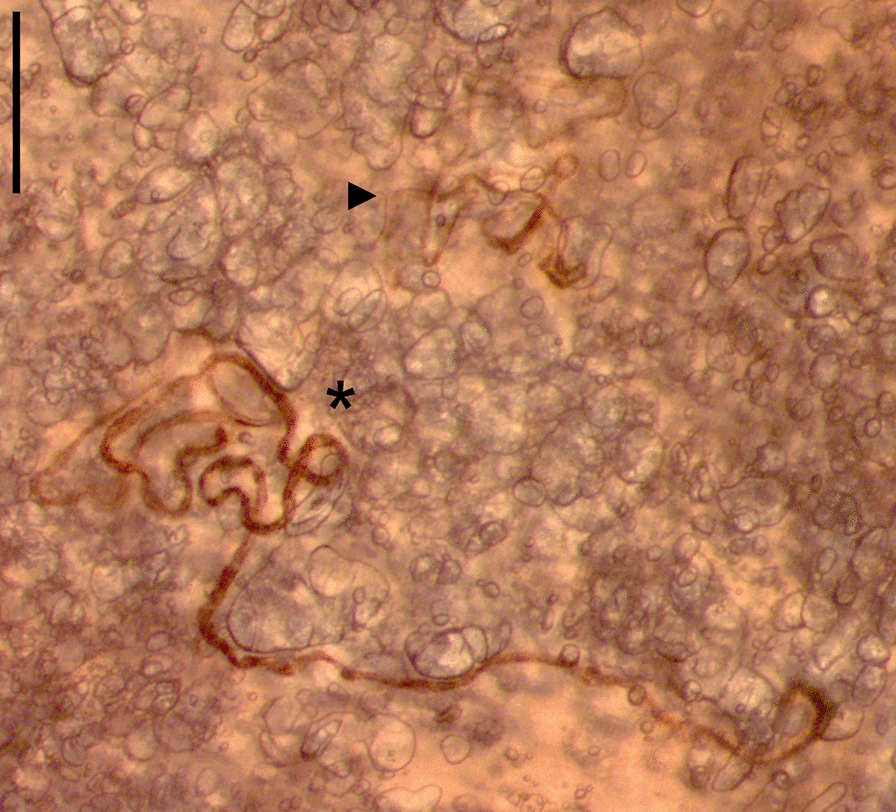
Section of a squeezed piece of lung with a female (*) and male (►) *Aelurostrongylus abstrusus*. The scale bar represents 500 µm

Heart ventricles, atria and outgoing blood vessels as well as trachea, main and accessory bronchi and large blood vessels of thawed hearts and lungs, respectively, were opened with scissors under 7× magnification (SLX-2; OPTIKA S.r.l., Ponteranica, Italy) for detection of inhabiting parasites. Subsequently, the lungs were cut into small pieces (maximum 3–4 mm), squeezed with a microscope slide into a petri dish and examined from both sides at 12.5× magnification (SLX-2; OPTIKA S.r.l., Ponteranica, Italy). Detected parasites as exemplarily shown in Fig. 2 were isolated and counted, followed by genus and, if possible, species identification according to morphological characteristics [8, 12, 13, 28–30].

### Molecular species identification of cardio-pulmonary parasites

To confirm morphological species determination, a total of 3–11 specimens of each detected species were subjected to DNA extraction. Total or partial parasites were disrupted with a pestle and lysed with 90 µl DirectPCR Lysis Reagent (DirectPCR^®^ Cell Lysis Reagent; Viagen Biotech Inc., Los Angeles, CA, USA) and 10 µl Proteinase K (20 mg/ml; Macherey-Nagel GmbH & Co. KG, Dueren, Germany) in case of *A. chabaudi* and *T. brevior* or half of each volume for the smaller *A. abstrusus* and *Capillaria* species. Samples were incubated at 55 °C for 14–16 h followed by 45 min at 85 °C to inactivate Proteinase K. DNA analyses were carried out by targeting the internal transcribed spacer (ITS) region of *A. abstrusus*, *T. brevior* and *A. chabaudi* in a 25-µl reaction set-up containing 0.5 µl DreamTaq DNA Polymerase (5.0 U/µl), 2.5 µl 10× DreamTaq Buffer (Thermo Fisher Scientific Inc., Waltham, MA, USA) and 0.5 µl dNTPs (10 mM each; Roti^®^-Mix PCR 3, Carl Roth GmbH+Co. KG, Karlsruhe, Germany). For molecular confirmation of *Capillaria* spp., the cytochrome c oxidase subunit 1 (Cox 1) and 18S rRNA gene were amplified in a 50 µl reaction volume with 0.25 µl DreamTaq DNA polymerase, 5.0 µl 10× DreamTaq buffer and 1.0 µl dNTPs. Detailed information on primers, template and cycling conditions is given in Table 1.

**Table 1:** PCR identification of detected cardio-pulmonary parasites

Species	Targeted DNA region	Primer pair	Template/reaction volume	Temperature profile	Amplicon size	References
*A. abstrusus*, *T. brevior*, *A. chabaudi*	ITS1-5.8S-ITS2	NC5, NC2 (0.2 µM each)^a^	5.0 µl^a^/25 µl^a^	95 °C^a^ for 3 min^a^; 40 ×^a^ 94 °C for 30 s, 55 °C for 30 s, 72 °C for 90 s^a^; 72 °C^a^ for 10 min^a^	~ 1400 bp	[60]
*Capillaria* spp.	Cox 1 mtDNA	COIFmod, COIRmod (1.0 µM each)	5.0 µl^a^/50 µl	95 °C for 3 min^a^; 40 × 94 °C for 30 s, 54 °C for 60 s, 72 °C for 60 s; 72 °C for 10 min	400 bp	[39]
	18S rRNA	18S 965, 18S 1573R (1.0 µM each)^a^	5.0 µl^a^/50 µl	95 °C^a^ for 3 min^a^; 40 ×^a^ 94 °C for 30 s, 53 °C^a^ for 30 s^a^, 72 °C^a^ for 60 s^a^; 72 °C for 10 min^a^	620 bp	[61]

^a^Modifications of PCR reference

Amplicons were visualised on GelRed^®^-supplemented (1:10,000; Biotium, Inc., Fremont, CA, USA) 1.5% agarose gels, and PCR products of the expected size were custom Sanger sequenced (Microsynth Seqlab GmbH, Goettingen, Germany). Obtained sequences were BLASTed against the NCBI standard databases and subsequently aligned with selected published reference sequences in Clone Manager (Version 9 Professional, Sci-Ed, Westminster, CO, USA). Sequences generated in the present study were deposited in GenBank under accession numbers OP452826-OP452827 (*A. abstrusus*), OP452837-OP452839 (*T. brevior*), OP452828-OP452836 **(***A. chabaudi*) and OP454031-OP454034 as well as OP453093-OP453095 (*Capillaria* spp.).

### Statistical analyses

R version 4.1.0 was used for statistical analyses [31]. For the prevalence of each parasite species, 95% confidence intervals were determined using the R function “binom.test”. Generalised linear models (GLMs) with binominal error structure were employed to assess the effects of the predictor variables ‘sex’, ‘age’, ‘nutritional condition’, ‘month of finding’ and ‘state of decomposition’ on the prevalence of the metastrongyloids *A. abstrusus*, *A. chabaudi* and *T. brevior.* To allow a suitable analysis, the factors ‘very good’ and ‘good’ as well as ‘bad’ and ‘very bad/cachectic’ and the factors ‘moderate fresh’ and ‘moderate rotten’ of the predictor variables ‘nutritional condition’ and ‘state of decomposition’, respectively, were combined. Additionally, infections with each of the other two metastrongyloids were included as predictors. As only four cats were infected with *Capillaria* spp.; this parasite was not considered separately. However, *Capillaria* spp. infection was included in a further model assessing the mentioned predictors (except for coinfections) on ‘total cardio-pulmonary parasite’ infection. Due to missing data, models were calculated for a subset of 103 wildcats. Each GLM was compared with a null model containing only the intercept using a likelihood ratio test.

## Results

### Key parameters of examined European wildcats

All European wildcats available for this study originated from the German federal state of Rhineland-Palatinate with sampling spots located in the districts of Palatinate (48.4%), Hunsrueck (19.5%), Westerwald (9.4%), Eifel (14.8%) and Taunus (3.1%) (Fig. 1). While 127 of the examined wildcats were morphologically or genetically identified as *F. silvestris*, one was a hybrid between *F. silvestris* and *F. catus*. The data on the studied animals listed in Table 2 were collected by the BUND and partly also by cooperating institutions during dissection. Due to a proceeded state of decay or incomplete carcasses, sex determination was not possible for 14 wildcats (10.9%) [26]; the others split into 68 males (53.1%) and 46 females (35.9%). Age determination revealed mainly adults (> 25 months, 53.9%) and the nutritional condition of most wildcats was very good or good (62.5%). Most animals were found in the autumn months, i.e. September (13.3%), October (9.4%) and November (20.3%). The state of decomposition was classified predominantly as moderate fresh or moderate rotten (59.4%) and the predominant cause of death was trauma (93.8%), mostly due to road traffic accidents.

**Table 2:** Key data of the examined wildcat specimens available for the study (*n* = 128)

	Number of wildcats (%)
Sex	
Male	68 (53.1)
Female	46 (35.9)
Not determinable	14 (10.9)
Age	
Adult (> 25 months)	69 (53.9)
Subadult (11–24 months)	19 (14.8)
Immature (5–10 months)	32 (25.0)
Juvenile (< 4 months)	7 (5.5)
Not determinable	1 (0.8)
Nutritional condition	
Very good	38 (29.7)
Good	42 (32.8)
Moderate	29 (22.7)
Bad	2 (1.6)
Very bad/cachectic	9 (7.0)
Not determinable	8 (6.3)
Month of finding	
January	12 (9.4)
February	8 (6.3)
March	12 (9.4)
April	7 (5.5)
May	6 (4.7)
June	6 (4.7)
July	7 (5.5)
August	6 (4.7)
September	17 (13.3)
October	12 (9.4)
November	26 (20.3)
December	6 (4.7)
Data not available	3 (2.3)
Region of origin	
Eifel	19 (14.8)
Hunsrueck	25 (19.5)
Palatinate	62 (48.4)
Taunus	4 (3.1)
Westerwald	12 (9.4)
Data not available	6 (4.7)
State of decomposition	
Fresh	41 (32.0)
Moderate fresh	56 (43.8)
Moderate rotten	20 (15.6)
Proceeded rotten	11 (8.6)
(Suspected) cause of death	
Trauma	120 (93.8)
Cachexia	1 (0.8)
Diarrhoea	1 (0.8)
Unknown	6 (4.7)

The data were collected by the BUND and partly also by cooperating institutions during dissection

### Examination of lungs and hearts

**Fig. 3:**
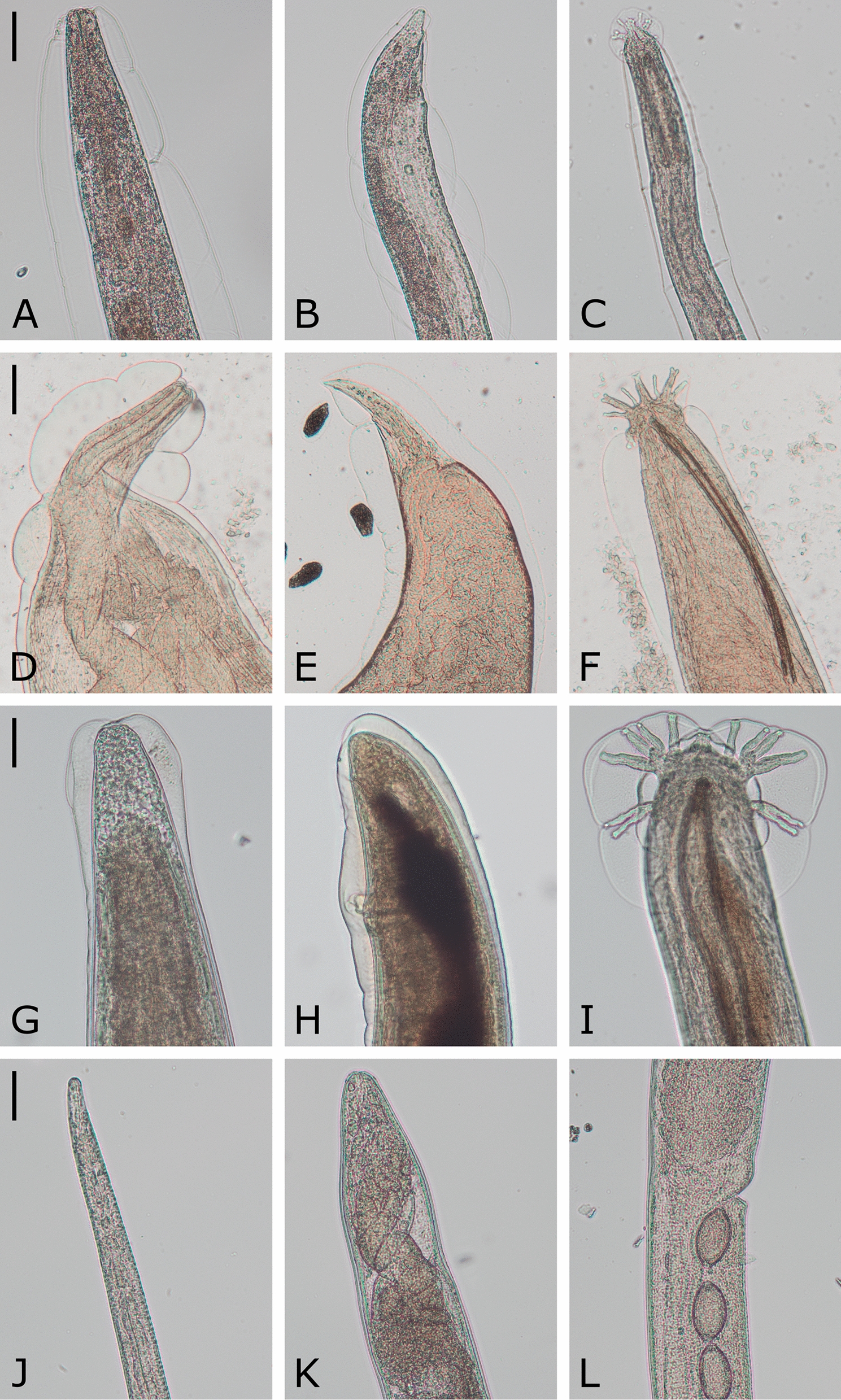
Cardio-pulmonary parasites isolated from heart and lung tissue of *Felis silvestris*. Representative specimens of *Aelurostrongylus abstrusus* (**A**–**C**; **A**: female anterior end, **B**: female posterior end, **C**: male posterior end), *Troglostrongylus brevior* (**D**–**F**; **D**: male anterior end, **E**: female posterior end, **F**: male posterior end), *Angiostrongylus chabaudi* (**G**–**I**; **G**: female anterior end, **H**: female posterior end, **I**: male posterior end) and *Capillaria* spp. (**J**–**L**; **J**: female anterior end, **K**: female posterior end, **L**: vulva region) are depicted. Scale bars represent 50 µm (*A. abstrusus, A. chabaudi*, *Capillaria* spp.) or 100 µm (*T. brevior*)

**Fig. 4:**
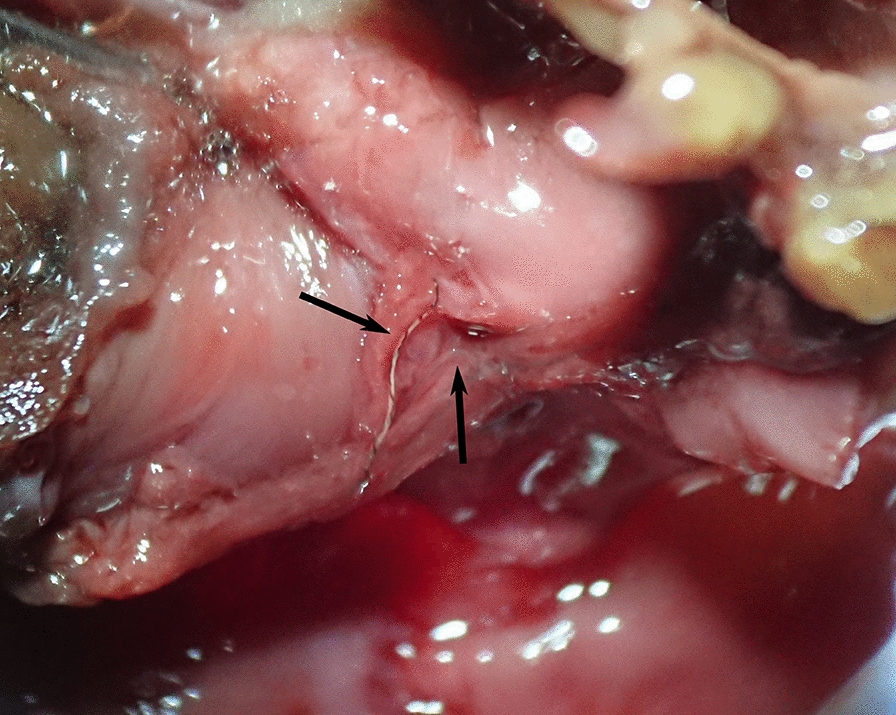
*Angiostrongylus chabaudi* (arrows) in the right atrium of an opened heart

In total, 70.3% of the wildcats harboured cardio-pulmonary parasites. Four different nematodes were detected, with *A. chabaudi* representing the most frequent species (53.1%) followed by *A. abstrusus* (42.2%) and *T. brevior* (31.3%), while *Capillaria* spp. were rather rare (3.1%). Detailed data are listed in Table 3 and representative specimens are pictured in Fig. 3 and Additional file 2. *Aelurostrongylus abstrusus* were mainly detected in squeezed lung pieces as they were firmly attached to the lung tissue. Besides the smaller size in general, males had a smaller bursa and shorter spicules and females had a shorter distance between anus and vulva (Fig. 3B, C) than *A. chabaudi* (Fig. 3H, I). *Angiostrongylus chabaudi* were not only found during squeezing but also frequently during opening of the large pulmonary blood vessels. The anterior ends of *A. chabaudi* carried the typical cephalic vesicle (Fig. 3G), which was absent in *A. abstrusus* (Fig. 3A). While *A. abstrusus*, *T. brevior* and *Capillaria* spp. were solely detected in the lung, *A. chabaudi* was also found in the heart (20.7%), as exemplarily shown in Fig. 4. In 20.6% (14/68) of the *A. chabaudi*-positive wildcats, worms were detected in both organs, while in 66.2% (45/68) worms were found only in the lungs and in 13.2% (9/68) only in the hearts. Of 12 *A. chabaudi*-positive wildcats, the heart was not available for examination. Furthermore, 20 lungs and 23 hearts were incomplete and therefore could only be partially examined. However, the impact on prevalence was small, at least for the lungs (partial lungs: 70.0% [14/20] vs. complete lungs: 70.4% [76/108]; partial hearts: 13.0% [3/23] vs. complete hearts: 22.7% [20/88]).

**Table 3:** Prevalence and infection intensities of cardio-pulmonary parasites in European wildcats from Germany (*n* = 128)

	Positive wildcats	Prevalence (%)	95% CI	Infection intensity			
				Min	Max	Mean	Median
Infections per parasite species (*n* = 128 wildcats)							
*Aelurostrongylus abstrusus*	54	42.2	33.5–51.2	1	167	17.3	5.5
*Troglostrongylus brevior*	40	31.3	23.4–40.0	1	26	4.9	2.0
*Angiostrongylus chabaudi* (lungs and hearts)	68	53.1	44.1–62.0	1	58	6.6	3.0
* A. chabaudi* (lungs only)	59	46.1	37.2–55.1	1	50	6.7	3.0
* A. chabaudi* (hearts only)	23	20.7^a^	13.6–29.5	1	8	2.4	1.0
*Capillaria* spp.	4	3.1	0.9–7.8	1	1	1.0	1.0
Total	90	70.3	61.6–78.1	1	167	17.6	7.5
Single and coinfections (*n* = 90 infected wildcats)			Prevalence (%) [95% CI] in all wildcats (*n* = 128)				
*A. abstrusus*	8	8.9	6.3 [2.7–11.9]	1	167	38.5	10.5
*T. brevior*	9	10.0	7.0 [3.3–12.9]	1	4	2.0	2.0
*A. chabaudi*	15	16.7	11.7 [6.7–18.6]	1	20	5.7	3.0
*A. abstrusus* + *T. brevior*	5	5.6	3.9 [1.3–8.9]	2	50	14.4	7.0
*A. abstrusus* + *A. chabaudi*	23	25.6	18.0 [11.7–25.7]	2	103	23.3	11.0
*T. brevior* + *A. chabaudi*	11	12.2	8.6 [4.4–14.9]	2	59	17.0	15.0
*A. chabaudi* + *Capillaria* spp.	1	1.1	0.8 [0.0–4.3]	2	2	2.0	2.0
*A. abstrusus* + *T. brevior* + *A. chabaudi*	15	16.7	11.7 [6.7–18.6]	5	86	23.8	16.0
*A. abstrusus* + *A. chabaudi* + *Capillaria* spp.	3	3.3	2.3 [0.5–6.7]	4	10	7.0	7.0

CI, confidence interval

^a^*n* = 111

Overall, a total of 35.6% (32/90) of the positive wildcats were single infected with *A. abstrusus* (8.9% [8/90]), *T. brevior* (10.0% [9/90]) or *A. chabaudi* (16.7% [15/90]). Coinfections with up to three different lungworm species were observed in 64.4% (58/90) of the wildcats (Table 3). *Aelurostrongylus abstrusus* and *A. chabaudi* were the most frequently detected double infection (25.6% [23/90]), followed by *T. brevior* and *A. chabaudi* (12.2% [11/90]), *A. abstrusus* and *T. brevior* (5.6% [5/90]) and *A. chabaudi* and *Capillaria* spp. (1.1% [1/90]). Triple infections were observed for *A. abstrusus*, *T. brevior* and *A. chabaudi* (16.7% [15/90]) as well as *A. abstrusus*, *A. chabaudi* and *Capillaria* spp. (3.3% [3/90]).

**Fig. 5:**
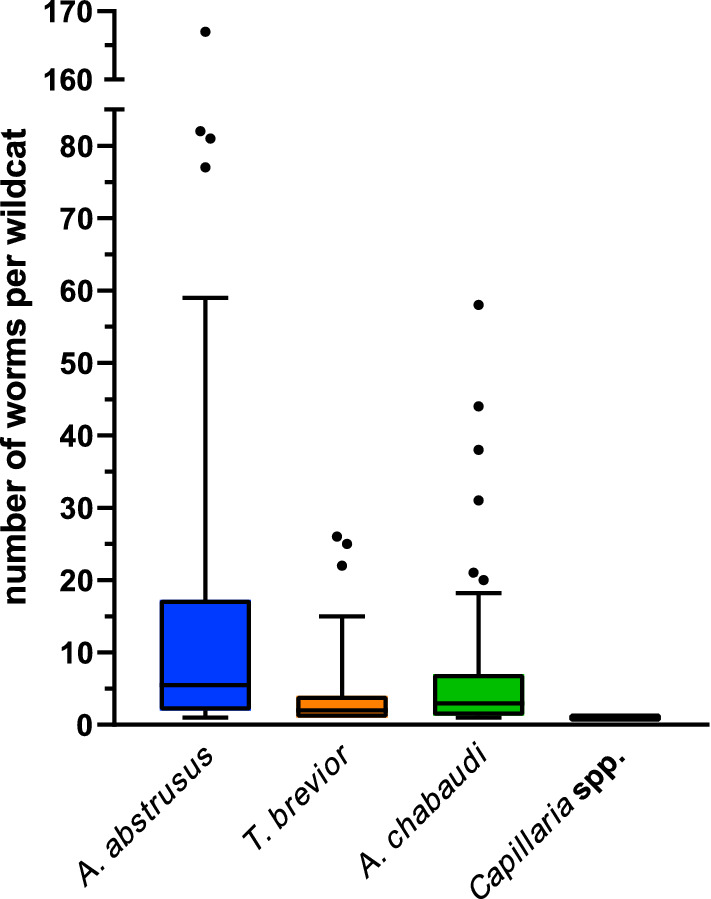
Infection intensities in *Felis silvestris* harbouring *Aelurostrongylus abstrusus* (*n* = 54), *Troglostrongylus brevior* (*n* = 40), *Angiostrongylus chabaudi* (*n* = 68) and *Capillaria* spp. (*n* = 4). Boxes extend from the 25th to the 75th percentile, with a line at the median and whiskers extending the 10th and 90th percentile, while dots represent individual data points beyond these percentiles

Infection intensities ranged from 1 to 167 cardio-pulmonary nematodes per wildcat, with 1–167 specimens of *A. abstrusus*, 1–26 of *T. brevior*, 1–58 of *A. chabaudi* and one *Capillaria* specimen each. More detailed data on infection intensities are given in Table 3 and Fig. 5.

### Molecular species identification

PCR cycling and Sanger sequencing of 3–11 worms per species resulted in sequences with appropriate quality of two *A. abstrusus*, three *T. brevior*, nine *A. chabaudi* and four *Capillaria* specimens (4× 18S rRNA amplicons; 3× Cox 1 amplicons). Sequence comparison of *A. abstrusus*, *T. brevior* and *A. chabaudi* amplicons showed 99.4–100% identity (query cover: 100%) with ITS sequences published in NCBI GenBank [acc. nos. KX518353 (*A. abstrusus*), KM506759 (*T. brevior*), KM979214 (*A. chabaudi*)]. The obtained 18S rRNA *Capillaria* spp. sequences revealed 100% identity (query cover: 100%) with *C. aerophila* isolated from red foxes in Switzerland and Italy (acc. nos. JX411622-JX411631) and 99.8% identity (query cover: 100%) with *Capillaria boehmi* from red foxes (*Vulpes vulpes*) in Italy (acc. nos. KC341986-KC341987). The three received Cox 1 mtDNA sequences differed in 1–4 nucleotides among each other. They showed 98.5–100% identity (query cover: 100%) with a *Capillaria* sp. sequence derived from a bronchoalveolar lavage of a cat in Australia (acc. no. MH665363), 83.8–85.3% identity (query cover: 100%) with *C. aerophila* from red foxes in Switzerland and Italy (acc. nos. KC341988-KC341991), 83.5–85.1% identity (query cover: 100%) with *C. aerophila* from cats and dogs in Italy (acc. nos. JQ905052-JQ905059) and 82.5–85.5% identity (query cover: 100%) with *C. boehmi* from red foxes and a dog in Italy and Norway (acc. nos. KC341992, KR186213-KR186215). Slightly lower identities with *Capillaria gastrica* from an Australian swamp rat (*Rattus lutreolus*) in Tasmania (81.3–82.0%, query cover: 100%, acc. no. AJ288163) and *Capillaria hepatica* from a European water vole (*Arvicola terrestris*) in Switzerland (80.8–81.6%, query cover: 100%, acc. no. KC355434) were found.

Overall, molecular species identification confirmed previous morphological diagnoses at species (*A. abstrusus*, *T. brevior*, *A. chabaudi*) or genus level (*Capillaria* spp.).

### Statistical analyses

The individual prevalences of each cardio-pulmonary parasite species, total infections and coinfections for each predictor variable in the GLM-analysed subset of 103 *F. silvestris* are listed in Additional file 1. Modelling indicated a significant association of *A. abstrusus* and *A. chabaudi* infection (Table 4). In fact, animals infected with *A. abstrusus* had approximately five times higher odds of also harbouring *A. chabaudi* (and vice versa) as compared to *A. abstrusus*-negative animals. Furthermore, a significant effect of age was found for *A. abstrusus* and *T. brevior* (Table 4) as well as for cardio-pulmonary parasite infections in total (Table 5). The total prevalence was higher in adult (84.9% [45/53]) than in subadult (58.8% [10/17]; *P* = 0.045) and juvenile individuals (28.6% [2/7]; *P* = 0.009), and *A. abstrusus* occurred significantly more often in adults (56.6% [30/53]) than in subadults (17.6% [3/17]; *P* = 0.022). In contrast, *T. brevior* was more frequent in immature (50.0% [13/26]; *P* = 0.012) than in adult wildcats (34.0% [18/53]). In addition, the *T. brevior* prevalence was significantly higher in males (38.7 [24/62]; *P* = 0.030) than in females (29.3% [12/41]) as well as in animals in bad/cachectic nutritional condition (66.7% [6/9]; *P* = 0.014) than in those in very good/good condition (36.2% [25/69]). Furthermore, a significant effect of the state of decomposition was found regarding detection of total cardio-pulmonary parasite infections as well as *A. abstrusus* and *T. brevior*. Significantly more total infections were observed in fresh (87.1% [27/31]) than in proceeded rotten wildcats (42.9% [3/7]; *P* = 0.023). Detection of *A. abstrusus* was also more common in fresh (67.7% [21/31]) than in moderate fresh/moderate rotten cadavers (35.4% [23/65]; *P* = 0.017). In contrast, *T. brevior* was detected significantly more often in moderate fresh/moderate rotten (38.5% [25/65]; *P* = 0.019) than in fresh animals (25.8% [8/31]).

**Table 4:** Results of GLM testing the influence of predictor variables on the prevalence of selected cardio-pulmonary parasite species in the subset of 103 *Felis silvestris*

	*Aelurostrongylus abstrusus*				*Troglostrongylus brevior*				*Angiostrongylus chabaudi*			
	Estimate	SE	*z*-value	*P*-value	Estimate	SE	*z*-value	*P*-value	Estimate	SE	*z*-value	*P*-value
Intercept	1.241	1.129	1.100	0.271	− 1.313	1.028	− 1.277	0.202	0.853	1.241	0.687	0.492
Sex (ref. male)												
Female	− 0.160	0.695	− 0.230	0.818	− 1.514	0.697	− 2.173	0.030*	1.153	0.613	1.881	0.060
Age (ref. adult)												
Subadult	− 2.462	1.077	− 2.286	0.022*	− 0.842	0.872	− 0.966	0.334	0.339	0.802	0.423	0.673
Immature	0.727	0.797	0.912	0.362	2.080	0.830	2.505	0.012*	− 1.438	0.748	− 1.921	0.055
Juvenile	− 1.390	1.410	− 0.986	0.324	− 1.244	1.602	− 0.777	0.437	− 1.600	1.180	− 1.356	0.175
Nutritional condition (ref. very good/good)												
Moderate	− 0.604	0.758	− 0.797	0.425	− 1.505	0.768	− 1.959	0.050*	0.914	0.701	1.304	0.192
Bad/cachectic	2.029	1.320	1.538	0.124	2.939	1.197	2.455	0.014*	0.543	1.152	0.472	0.637
Month of finding (ref. January)												
February	− 1.849	1.607	− 1.150	0.250	− 3.223	1.834	− 1.758	0.079	− 3.134	1.633	− 1.919	0.055
March	− 2.152	1.312	− 1.641	0.101	0.659	1.058	0.623	0.534	− 1.959	1.345	− 1.457	0.145
April	0.347	1.416	0.245	0.806	− 0.244	1.197	− 0.204	0.839	− 2.966	1.537	− 1.930	0.054
May	− 2.352	1.818	− 1.294	0.196	− 18.646	1391.561	− 0.013	0.989	− 2.997	1.614	− 1.857	0.063
June	17.601	1489.149	0.012	0.991	− 0.689	2.452	− 0.281	0.779	− 3.879	2.012	− 1.928	0.054
July	0.620	1.636	0.379	0.705	− 0.384	1.447	− 0.266	0.791	− 2.565	1.618	− 1.585	0.113
August	− 2.264	1.722	− 1.315	0.189	− 0.791	1.515	− 0.522	0.601	− 2.266	1.792	− 1.265	0.206
September	0.062	1.309	0.048	0.962	− 1.955	1.276	− 1.531	0.126	− 1.946	1.366	− 1.425	0.154
October	− 1.012	1.257	− 0.805	0.421	− 1.462	1.201	− 1.217	0.224	− 1.540	1.406	− 1.095	0.273
November	− 1.181	1.019	− 1.159	0.246	− 1.182	0.883	− 1.338	0.181	− 1.070	1.197	− 0.894	0.371
December	− 2.972	1.866	− 1.592	0.111	− 0.265	1.321	− 0.201	0.841	− 1.735	1.532	− 1.133	0.257
State of decomposition (ref. fresh)												
Moderate fresh/moderate rotten	− 1.791	0.752	− 2.383	0.017*	1.710	0.729	2.345	0.019*	0.050	0.648	0.076	0.939
Proceeded rotten	− 1.051	1.345	− 0.781	0.435	1.539	1.229	1.252	0.211	− 1.494	1.276	− 1.171	0.242
Coinfection												
With *A. abstrusus*					− 0.660	0.660	− 1.000	0.317	1.580	0.592	2.670	0.008*
With *T. brevior*	− 0.278	0.635	− 0.438	0.662					0.997	0.618	1.613	0.107
With *A. chabaudi*	1.670	0.644	2.591	0.010*	1.312	0.673	1.951	0.051				

The full models were significantly different from a null model containing only the intercept (*A. abstrusus*: χ^2^ = 50.8, *df* = 21, *P* < 0.001; *T. brevior*: χ^2^ = 38.1, Df = 21, *P* = 0.013; *A. chabaudi*: *χ*^2^ = 35.7, *df* = 21, *P* = 0.024)

SE, standard error

^*^Significant *P*-values (≤ 0.05)

**Table 5:** Results of GLM testing the influence of predictor variables on the prevalence of cardio-pulmonary parasites (all species included) in the subset of 103 *Felis silvestris*

	Cardio-pulmonary parasites			
	Estimate	SE	*z*-value	*P*-value
Intercept	2.977	1.220	2.439	0.015*
Sex (ref. male)				
Female	0.245	0.649	0.378	0.706
Age (ref. adult)				
Subadult	−1.708	0.851	−2.008	0.045*
Immature	−0.137	0.867	−0.158	0.874
Juvenile	−3.199	1.232	−2.598	0.009*
Nutritional condition (ref. very good/good)				
Moderate	0.020	0.751	0.026	0.979
Bad/cachectic	2.710	1.632	1.660	0.097
Month of finding (ref. January)				
February	−2.500	1.478	−1.692	0.091
March	−0.469	1.462	−0.321	0.748
April	0.423	1.667	0.253	0.800
May	−2.580	1.626	−1.586	0.113
June	14.816	1383.132	0.011	0.991
July	0.075	2.152	0.035	0.972
August	−2.170	1.768	−1.228	0.220
September	−0.017	1.536	−0.011	0.991
October	−2.011	1.384	−1.453	0.146
November	−0.163	1.286	−0.127	0.899
December	−0.520	1.621	−0.321	0.748
State of decomposition (ref. fresh)				
Moderate fresh/moderate rotten	−0.628	0.769	−0.817	0.414
Proceeded rotten	−2.797	1.234	−2.266	0.023*

The full model was significantly different from a null model containing only the intercept (*χ*^2^ = 33.9, *df* = 19, *P* = 0.019)

SE, standard error

^*^Significant *P*-values (≤ 0.05)

No statistically significant seasonal differences were found when comparing the months of finding.

## Discussion

Almost three quarters of the wildcats (70.3%) were infected with at least one cardio-pulmonary nematode species, confirming previous reports on the presence of *A. abstrusus*, *T. brevior, A. chabaudi* and *Capillaria* spp. in *F. silvestris* in Germany [24, 25].

In Europe, the prevalences of lungworm species vary between different studies. While the present study revealed a prevalence of 42.2% for *A. abstrusus*, Steeb [25] detected nematode stages in 27 of 85 (31.8%) histologically examined lungs of *F. silvestris* from different parts of Germany and Luxembourg, of which 21 (24.7%) were infected with larval stages of *A. abstrusus*. In Greece, infection rates were comparable with a prevalence of 43.5% in necropsied wildcats [22], whereas the prevalence in Romania was lower with 4.3% [23]. In Italy, prevalences ranged from 0.0 to 62.5% [19, 20]. This high variance in Italian prevalences and the low prevalence in Romania may be explained by methodological differences. Veronesi et al. [20] dissected damaged lung tissue with forceps resulting in 62.5% *A. abstrusus*-positive lungs, while Falsone et al. [19] and Deak et al. [23] examined rinses of squeezed lung tissue with no or only low detection of *A. abstrusus*, respectively.

Although *T. brevior* has already been detected in European wildcats in Germany, data on its prevalence have not been available so far [25]. The *T. brevior* prevalence of 31.3% found in the present study was higher than expected, as this parasite was previously assumed to play a more important role in southern rather than in more northern European countries [32]. In Southern Europe, lower, similar or higher prevalences ranging between 14.9 and 71.4% were observed for *F. silvestris* in Romania, Greece and Italy, respectively [19, 20, 22, 23]. In domestic cats, lower rates of 1.4–14.2% were reported in Bulgaria, Spain and various regions of Italy based on faecal examination [33]. The rather high prevalence detected in the current study could be due to an increased occurrence in Germany in recent years due to climate change, as *T. brevior* develops most rapidly at 22–27 °C in intermediate hosts such as *Helicella* spp. [7]. Moreover, the increasing distribution of *T. brevior*’s natural host, namely *F. silvestris*, might be positively related to the occurrence of this parasite[34]. Another possible hypothesis is that *T. brevior* might have already been endemic in Germany for a longer period, but was usually misdiagnosed in routine faecal diagnosis of domestic cats, probably mainly in clinical settings, because of the high similarity of L1 to those of *A. abstrusus* [8, 16].

Compared to other feline cardio-pulmonary nematodes, *A. chabaudi* has been neglected for a long time. More than 50 years after its first description in a European wildcat [15], this parasite was “rediscovered” in a domestic cat in Italy in 2014 [35]. Since then, further cases of infected wildcats in Romania and Bosnia and Herzegovina have been reported [36, 37]. Moreover, prevalences of 6.3–56.5% in dissected wildcats were found in Italy, Romania and Greece, respectively [20, 22, 23]. The prevalence of 53.1% determined in the present study may be slightly underestimated because the hearts of five wildcats negative for *A. chabaudi* in their lungs were not available for examination. However, the prevalence rate is similar to that observed in Greece and higher than that in Italy. In both the present and the Greek study [22], *A. chabaudi* was the most common cardio-pulmonary parasite and was correspondingly detected more frequently than its much better known relative *A. abstrusus*. Due to its frequent occurrence, more attention should be paid to *A. chabaudi*, and further studies on its life cycle are needed to unravel potential transmission cycles to wildcats (and domestic cats [35, 38]) and its pathogenicity.

The determined prevalence of *Capillaria* spp. in the examined wildcats was 3.1%. In contrast, Krone et al. [24] detected *C. aerophila* in 13.3% (2/15) *F. silvestris* from the German federal states North Rhine-Westphalia, Rhineland-Palatinate and Saarland. In Italy, Greece and Romania, *C. aerophila* prevalence ranges from 18.8 to 34.0% [19, 20, 22, 23]. A possible explanation for the low prevalence in the present study could be the examination method, where additional rinsing of the opened trachea and bronchi might have resulted in higher recovery, as *Capillaria* specimens may have been missed in the mucosa.

Unfortunately, species identification of *Capillaria* spp. by sequencing 18S rRNA and Cox 1 amplicons was not feasible. While the 18S rRNA shows a high degree of conservation, allowing reliable genus determination [39], the mitochondrial Cox 1 gene harbours high intraspecific polymorphisms as observed in *C.* *aerophila* isolated from dogs and cats in Italy, where eight *C. aerophila* haplotypes were observed with differences ranging from 0.4 to 5.5% [18]. The three Cox 1 sequences obtained in the present study differed by 14.9–16.5% from the above-mentioned *C. aerophila* sequences, indicating that the found *Capillaria* sp. represents at least a new haplotype, if not a new species. The parasites’ worldwide spread and wide range of different hosts including cats, dogs and many different wild mammals could explain the existence of highly diverse haplotypes. Unfortunately, the haplotypes of *C. aerophila* are unknown in both wildcats and domestic cats in Europe, with the exception of Italy [18, 20]. However, the observed high genetic distance to *C. aerophila*, comparable to that to *C. boehmi*, rather implies that the species found here is not *C. aerophila*. *Capillaria boehmi*, another capillariid of low host specificity [40] that occurs in the respiratory tract of carnivores (mainly foxes, mustelids and dogs [41]), can not only be deferred because of its low nucleotide identity, but furthermore excluded because of its specific localisation in the host [40]. While *C. boehmi* parasitises the upper respiratory tract, i.e. nasal cavities and sinuses [41], the *Capillaria* specimens in this study were detected exclusively in the trachea and bronchi. Interestingly, these detected specimens showed a high degree of Cox 1 nucleotide identity (98.5–100%) with a *Capillaria* specimen from an Australian cat (GenBank acc. no. MH665363), which merits further investigations.

About one-third of the infected wildcats showed single infections, whereas approximately two-thirds harboured two or three cardio-pulmonary nematode species. The most frequently detected coinfection was that of *A. abstrusus* and *A. chabaudi*, which affected about a quarter of the positive wildcats. Thus, this infection was also the most frequent overall, regardless of whether single or coinfections were considered. The second most common infection was a single infection with *A. chabaudi* and a triple infection with *A. abstrusus*, *A. chabaudi* and *T. brevior*, each with equal percentages. However, unlike the coinfection of *A. abstrusus* and *A. chabaudi*, these were not statistically significantly associated. In other studies from Italy and Romania, coinfections with 2–4 different species accounted for about one-third of all infections. The most frequent coinfections were those of *A. abstrusus* and *T. brevior* and of *A. chabaudi* and *C. aerophila* in 20.0 and 13.8% of positive animals, respectively [20, 23].

Besides coinfections, the intensity of infection can have various effects on the health of a wildlife population [42]. In this study, infection intensities ranged from a single to up to 167 specimens. In most cases, however, the wildcats suffered only from mild infections with a few specimens of each parasite species. However, it has to be considered that experimental factors may have contributed to the low infection intensity, such as limited detection of partially decomposed worms or their outright loss due to decomposition. Furthermore, the *A. chabaudi* intensity may have been higher in the 12 animals in which the lungs were positive but no hearts were available for examination. Nevertheless, also other studies report comparably low infection intensities, with three *T. brevior* individuals found in a wildcat from Romania [43] and 9–12 *A. chabaudi* in *F. silvestris* from Bosnia and Herzegovina, Greece and Romania [12, 36, 37].

The prevalence of parasites in wildlife is influenced by many factors [42]. In the present study, sample condition as an indirect factor and all intrinsic factors tested (sex, age, nutritional condition) influenced the prevalence of at least one cardio-pulmonary nematode species, whereas the extrinsic factor ‘month of finding’ had no effect. The significantly higher prevalence of *A. abstrusus* and total cardio-pulmonary nematodes in fresh than in moderate fresh or rotten carcasses is attributable to the corresponding decomposition of the parasites, with the very thin *A. abstrusus* in particular becoming lytic and therefore difficult to detect. In contrast, *T. brevior* was detected significantly more often in moderate fresh/moderate rotten than in fresh individuals, indicating that a mild decomposition of host tissue does not affect the detection of larger parasites.

Regarding intrinsic factors, the significantly higher *T. brevior* prevalence in male than female wildcats may be explained by the immunosuppressive effect of testosterone [44] or behavioural differences. Compared to female *F. silvestris*, males require more prey and have a wider prey spectrum due to their larger size and home ranges [45], making them more likely to have higher infection rates. However, higher infection rates in males were not observed for other parasite species, indicating that sex-dependency for *T. brevior* might rather be a random effect. Moreover, the occurrence of not only male- but also female-biased parasitism, being favoured by e.g. immunosuppression during pregnancy and lactation, is frequently reported; therefore, the effect of sex on parasite infection is often controversially discussed [46]. Host age had a significant effect on the prevalence of *A. abstrusus* and total cardio-pulmonary parasite infections, which were more common in adult wildcats than in younger age groups. This observation was already reported for *A. abstrusus* in domestic cats [47]. An opposite age dependency was observed regarding *T. brevior* infection, where immature (5–10 months) animals were significantly more often infected than adults (> 25 months). In Italy, the same observation was made for domestic cats, with younger individuals (≤ 6 months and < 1 year, respectively) being more frequently infected than cats in older age groups [48, 49]. Age dependency might be mediated by differing lifespans of and immunity against the respective parasites. Clinical studies with experimentally infected domestic cats show that infections with *A. abstrusus* last for at least 25 weeks [50], whereas infections with *T. brevior* start to resolve after 6–9 weeks [51]. The long lifespan of *A. abstrusus* and the potentially impeded development of immunity may favour the accumulation of this parasite in the host, whereas the short lifespan of *T. brevior* and a presumed development of efficient immunity counteracts accumulation and reinfection, thus limiting its occurrence mainly to younger animals. Although the lifespan of the parasites and/or the immune response might differ between domestic cats and wildcats, the effect of nutritional condition, which was negatively correlated with the infection rate of *T. brevior*, with animals in bad/cachectic condition being more frequently infected than those in good/very good condition, also supports this hypothesis. For instance, a poor condition may favour parasite persistence due to a weakened immune response, while parasite infection itself can lead to the deterioration of body condition, for instance by damaging host tissue [42, 52]. To confirm this hypothesis, further studies on feline immunity to cardio-pulmonary nematode infections are needed.

Unfortunately, it is difficult to relate the determined parasite infection intensities to the wildcats’ health status as no further examinations could be performed and only few comparative data are available. Histological lung examination of single or coinfected wildcats revealed mild to severe pulmonary lesions and (broncho-)pneumonia attributed to *A. abstrusus*, *T. brevior* and/or *A. chabaudi* [20, 37]. Affected animals may also suffer clinically as shown for a wildcat infected with *A. abstrusus*, *T. brevior*, *A. chabaudi* and *C. aerophila*, which was hospitalised in very poor general condition with respiratory symptoms and severe lung lesions on radiograph [53]. Such reports, although rare, demonstrate the potential impact of cardio-pulmonary parasites on the health of individual European wildcats, especially when animals suffer from coinfections, which may exhibit increased pathogenicity compared to single infections [54]. In the present study, however, about two-thirds of the infected wildcats were coinfected with two to three cardio-pulmonary nematode species, but only a small proportion was in bad or cachectic condition, suggesting that such multiple infections alone are usually not causative of poor health and that other factors contribute to this condition.

While knowledge on wildcats is rather poor, the clinical impact of lungworm infections in domestic cats is more comprehensively described. In these, *A. abstrusus* and *T. brevior* can cause severe respiratory symptoms and *T. brevior* infections of kittens in particular can be fatal [11, 13, 55, 56]. Infections with *C. aerophila* may also result in bronchial disease with cough and wheezing [14]. In contrast, the effects of *A. chabaudi* infections in domestic cats are unknown to date. In one of the two published cases, no pathohistological examination of the road-killed cat was reported [35], and in the other case, the cat suffered from a mixed infection with *A. abstrusus* and *T. brevior* [57]. The necropsy of this cat, which showed severe respiratory distress and died shortly after admission, revealed no parasitological or pathological findings consistent with cat angiostrongylosis. Nevertheless, no conclusions about the (lack of) pathogenic potential of *A. chabaudi* in domestic cats can be drawn from a case with only two adult nematodes detected. Therefore, a potential emergence in domestic cats poses an unpredictable health impact [38].

Overall, there is a risk of spillover of feline cardio-pulmonary parasites at the wildlife-domestic animal interface. Not only do the different parasites share the same intermediate and paratenic hosts, e.g. small rodents, birds and other vertebrates [7–9], but these hosts, especially small rodents, are also preyed upon by both wild and domestic cats. Thus, indirect transmission from European wildcats to domestic cats and vice versa can be assumed [20, 24, 34]. The high prevalence of cardio-pulmonary nematodes in wildcats observed in the present study may favour their indirect transmission to domestic cats and pose a serious health threat; therefore, awareness of lungworm infection in domestic cats should be raised.

## Conclusions

Four different cardio-pulmonary parasites dominate in *F. silvestris* in Germany. While the metastrongyloids *A. abstrusus*, *T. brevior* and *A. chabaudi* were present in about one-third to one-half of the wildcats, the trichuroid *Capillaria* sp. was less common with a prevalence of only about 3%. *Angiostrongylus chabaudi* was the most frequently detected cardio-pulmonary nematode but is the least well studied, and its life cycle and pathogenicity should be further explored in future studies. Furthermore, our study showed an accumulation of *A. abstrusus* infections in adult wildcats, while *T. brevior* was significantly more often detected in immatures than adults. This could indicate, on the one hand, a lacking or insufficient development of immunity and, on the other hand, a rather efficient immunity, which calls for future studies on feline immunity to cardio-pulmonary nematode infections. Also, attention should be drawn to the epidemiological and health implications of coinfections, which accounted for about two-thirds of the infections. The impact of cardio-pulmonary nematodes on the health of individual wildcats is difficult to assess from our study, which examined dead found animals. Nevertheless, since most wildcats appeared to be healthy because of their mostly good or very good nutritional status, infections with cardio-pulmonary nematodes appear to play only a minor role in the overall health of the German wildcat population. However, because of the presumed spillover to domestic cats, veterinarians should pay more attention to these parasites and consider them as a differential diagnosis in cats with cardio-pulmonary signs.

## Supplementary Information

**Additional file 1: Table S1.** Nutritional condition classification of *Felis silvestris* carcasses based on fat deposits. **Table S2.** Prevalences of cardio-pulmonary parasites and coinfections for each predictor variable in the GLM-analysed subset of 103 *F. silvestris*.

**Additional file 2: Figure S1.** Feline cardio-pulmonary nematodes isolated from heart and lung tissues of *Felis silvestris*.

## Data Availability

Data supporting reported results is contained within the article and its additional files. Generated sequences were deposited in GenBank under accession numbers OP452826-OP452827 (*A. abstrusus*), OP452837-OP452839 (*T. brevior*), OP452828-OP452836 (*A. chabaudi*) and OP454031-OP454034 and OP453093-OP453095 (*Capillaria* spp).

## Abbreviations

BLAST: Basic Local Alignment Search Tool
BUND: *Bund fuer Umwelt und Naturschutz Deutschland*
Cox 1: Cytochrome c oxidase subunit 1
DNA: Deoxyribonucleic acid
dNTPs: Deoxynucleotide triphosphates
GLM: Generalised linear model
ITS: Internal transcribed spacer
IUCN: International Union for Conservation of Nature
L1: First-stage larvae
L3: Third-stage larvae
mtDNA: Mitochondrial DNA
NCBI: National Center for Biotechnology Information
PCR: Polymerase chain reaction
RNA: Ribonucleic acid
rRNA: Ribosomal RNA
