# Supplementary material for: Cardio-pulmonary parasites of the European wildcat (Felis silvestris) in Germany
Source: Parasit Vectors. 2022 Dec 5;15:452. doi: 10.1186/s13071-022-05578-z (PMC9724372; doi:10.1186/s13071-022-05578-z)
Supplement: Supplementary file 1 — Additional file 1: Table S1. Nutritional condition classification of Felis silvestris carcasses based on fat deposits. Table S2. Prevalences of cardio-pulmonary parasites and coinfections for each predictor variable in the GLM-analysed subset of 103 F. silvestris. [file 13071_2022_5578_MOESM1_ESM.docx]

Table S1 Nutritional condition classification of Felis silvestris carcasses based on fat deposits.

| Nutritional status | Subcutaneous and visceral fat | Kidney and coronary fat |
| --- | --- | --- |
| Very good | Both in plates or | Both present |
|  | One in plates, one in strands |  |
| Good | Both in strands or | Both present |
|  | One in strands, one isolated |  |
| Moderate | Both isolated or | Both present |
|  | One isolated or in strands, one absent |  |
| Bad | Both absent | Both present |
| Very bad/cachectic | Both absent | One or both absent |

Table S2 Prevalences of cardio-pulmonary parasites and coinfections for each predictor variable in the GLM-analysed subset of 103 *F. silvestris*.

|  |  | *A. abstrusus* | | *T. brevior* | | *A. chabaudi* | | *Capillaria* spp. | | Total parasites | |
| --- | --- | --- | --- | --- | --- | --- | --- | --- | --- | --- | --- |
| Predictor variable | Total wildcats | Positives | Prevalence (%) | Positives | Prevalence (%) | Positives | Prevalence (%) | Positives | Prevalence (%) | Positives | Prevalence (%) |
| Sex |  |  |  |  |  |  |  |  |  |  |  |
| male | 62 | 27 | 43.5 | 24 | 38.7 | 32 | 51.6 | 2 | 3.2 | 45 | 72.6 |
| female | 41 | 19 | 46.3 | 12 | 29.3 | 27 | 65.9 | 2 | 4.9 | 32 | 78.0 |
| Age |  |  |  |  |  |  |  |  |  |  |  |
| adult | 53 | 30 | 56.6 | 18 | 34.0 | 36 | 67.9 | 2 | 3.8 | 45 | 84.9 |
| subadult | 17 | 3 | 17.6 | 4 | 23.5 | 9 | 52.9 | 2 | 11.8 | 10 | 58.8 |
| immature | 26 | 12 | 46.2 | 13 | 50.0 | 12 | 46.2 | 0 | 0.0 | 20 | 76.9 |
| juvenile | 7 | 1 | 14.3 | 1 | 14.3 | 2 | 28.6 | 0 | 0.0 | 2 | 28.6 |
| Nutritional condition |  |  |  |  |  |  |  |  |  |  |  |
| very good/good | 69 | 32 | 46.4 | 25 | 36.2 | 41 | 59.4 | 1 | 1.4 | 53 | 76.8 |
| moderate | 25 | 7 | 28.0 | 5 | 20.0 | 12 | 48.0 | 2 | 8.0 | 16 | 64.0 |
| bad/cachectic | 9 | 7 | 77.8 | 6 | 66.7 | 6 | 66.7 | 1 | 11.1 | 8 | 88.9 |
| Month of finding |  |  |  |  |  |  |  |  |  |  |  |
| January | 11 | 8 | 72.7 | 6 | 54.5 | 10 | 90.9 | 0 | 0.0 | 10 | 90.9 |
| February | 6 | 1 | 16.7 | 1 | 16.7 | 1 | 16.7 | 0 | 0.0 | 2 | 33.3 |
| March | 10 | 3 | 30.0 | 4 | 40.0 | 6 | 60.0 | 0 | 0.0 | 8 | 80.0 |
| April | 7 | 4 | 57.1 | 4 | 57.1 | 3 | 42.9 | 0 | 0.0 | 6 | 85.7 |
| May | 5 | 1 | 20.0 | 0 | 0.0 | 2 | 40.0 | 0 | 0.0 | 3 | 60.0 |
| June | 2 | 2 | 100.0 | 1 | 50.0 | 1 | 50.0 | 1 | 50.0 | 2 | 100.0 |
| July | 6 | 4 | 66.7 | 2 | 33.3 | 4 | 66.7 | 1 | 16.7 | 5 | 83.3 |
| August | 5 | 2 | 40.0 | 3 | 60.0 | 2 | 40.0 | 0 | 0.0 | 3 | 60.0 |
| September | 12 | 6 | 50.0 | 2 | 16.7 | 6 | 50.0 | 1 | 8.3 | 9 | 75.0 |
| October | 9 | 4 | 44.4 | 3 | 33.3 | 5 | 55.6 | 0 | 0.0 | 5 | 55.6 |
| November | 24 | 10 | 41.7 | 8 | 33.3 | 16 | 66.7 | 1 | 4.2 | 20 | 83.3 |
| December | 6 | 1 | 16.7 | 2 | 33.3 | 3 | 50.0 | 0 | 0.0 | 4 | 66.7 |
| State of decomposition |  |  |  |  |  |  |  |  |  |  |  |
| fresh | 31 | 21 | 67.7 | 8 | 25.8 | 21 | 67.7 | 1 | 3.2 | 27 | 87.1 |
| moderate fresh/ moderate rotten | 65 | 23 | 35.4 | 25 | 38.5 | 36 | 55.4 | 3 | 4.6 | 47 | 72.3 |
| proceeded rotten | 7 | 2 | 28.6 | 3 | 42.9 | 2 | 28.6 | 0 | 0.0 | 3 | 42.9 |
| Coinfection |  |  |  |  |  |  |  |  |  |  |  |
| with *A. abstrusus* | 46 |  |  | 18 | 39.1 | 35 | 76.1 | 3 | 6.5 |  |  |
| with *T. brevior* | 36 | 18 | 50.0 |  |  | 24 | 66.7 | 0 | 0.0 |  |  |
| with *A. chabaudi* | 59 | 35 | 59.3 | 24 | 40.7 |  |  | 4 | 6.8 |  |  |
| with *Capillaria* spp. | 4 | 3 | 75.0 | 0 | 0.0 | 4 | 100.0 |  |  |  |  |
