# Supplementary material for: Cardio-pulmonary parasites of the European wildcat (Felis silvestris) in Germany
Source: Parasit Vectors. 2022 Dec 5;15:452. doi: 10.1186/s13071-022-05578-z (PMC9724372; doi:10.1186/s13071-022-05578-z)
Supplement: Supplementary file 2 — Additional file 2: Figure S1. Feline cardio-pulmonary nematodes isolated from heart and lung tissues of Felis silvestris. [file 13071_2022_5578_MOESM2_ESM.docx]

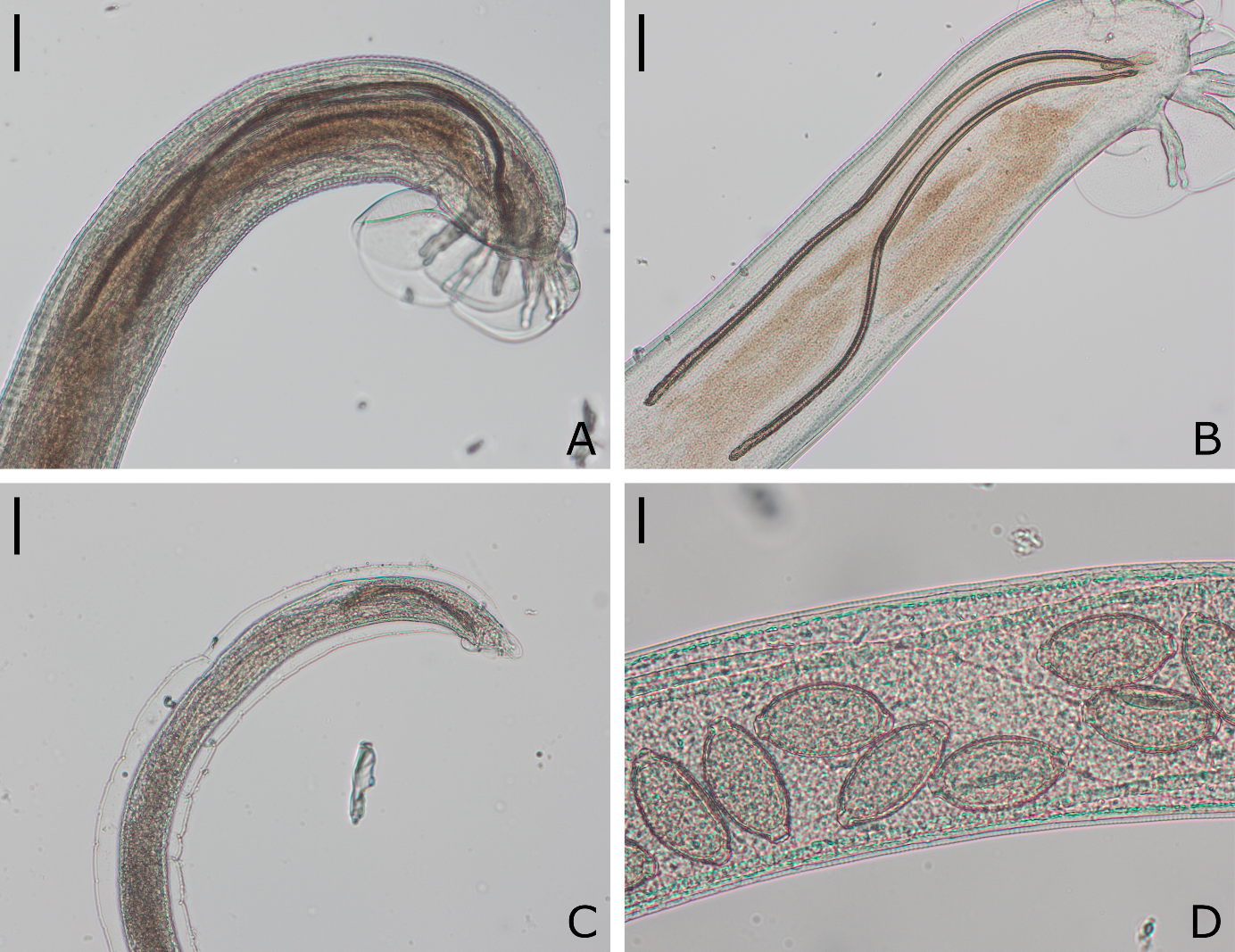


Figure S1: Feline cardio-pulmonary nematodes isolated from heart and lung tissues of Felis silvestris: Male Angiostrongylus chabaudi (A-B; A: lateral view of the posterior end, B: spicules), male Aelurostrongylus abstrusus (C: lateral view of the posterior end) and female Capillaria spp. (D: section of the uterus filled with eggs). Scale bars represent 50 µm (A. chabaudi, A. abstrusus) or 20 µm (Capillaria spp.).
